# Genetic Diversity of Toll-Like Receptors and Immunity to *M. leprae* Infection

**DOI:** 10.1155/2012/415057

**Published:** 2012-03-18

**Authors:** Bryan E. Hart, Richard I. Tapping

**Affiliations:** ^1^Department of Microbiology, University of Illinois, Urbana, IL 61801, USA; ^2^College of Medicine, University of Illinois, Urbana, IL 61801, USA

## Abstract

Genetic association studies of leprosy cohorts across the world have identified numerous polymorphisms which alter susceptibility and outcome to infection with *Mycobacterium leprae*. As expected, many of the polymorphisms reside within genes that encode components of the innate and adaptive immune system. Despite the preponderance of these studies, our understanding of the mechanisms that underlie these genetic associations remains sparse. Toll-like receptors (TLRs) have emerged as an essential family of innate immune pattern recognition receptors which play a pivotal role in host defense against microbes, including pathogenic strains of mycobacteria. This paper will highlight studies which have uncovered the association of specific TLR gene polymorphisms with leprosy or tuberculosis: two important diseases resulting from mycobacterial infection. This analysis will focus on the potential influence these polymorphic variants have on TLR expression and function and how altered TLR recognition or signaling may contribute to successful antimycobacterial immunity.

## 1. Introduction

*Mycobacterium leprae* is an evolutionarily ancient pathogen of historical and worldwide prevalence. Identified in 1873 by Gerhard Armauer Hansen as the causative agent of leprosy, these fastidious, intracellular bacilli have been studied extensively for their complex pathogenesis and host interactions. Cellular tropisms for *M. leprae* principally include tissue-resident macrophages, especially in the skin and upper respiratory tract, and Schwann cells of the peripheral nervous system. The intricate spectrum of clinical manifestations which exists for the disease is illustrated by two polar responses, tuberculoid or lepromatous leprosy, and various intermediate or borderline forms [1]. The tuberculoid manifestation is typically less severe and is characterized by lower leprosy burden and containment of bacilli by distinct granulomas. This form exhibits few hypopigmented skin lesions and scarce thickening of peripheral nerves, leading to loss of sensation in extremities and the skin. Lepromatous leprosy constitutes the opposite polar reaction to infection where high, diffuse bacterial load can cause extensive skin plaques, nodules and thickening, and numerous anesthetic zones due to pervasive peripheral nerve damage. 

 Surprisingly little genotypic variation exists between strains of *M. leprae*, a fact inconsistent with the high degree of variability in virulence and disease penetrance between individuals. This suggests that success of infection and leprosy progression rests in large part upon the patient's immune response and genetic complement. Strikingly, the variable spectrum of leprosy outcome correlates tightly with the balance of T-helper-cell-mediated immunity responding to* M. leprae* infection. A robust T_H_1 host response, featuring appreciable production of interferon-gamma (IFN*γ*) and interleukin-12 (IL-12) along with macrophage and CD4^+^ T cell activation, is typically represented in the contained, tuberculoid form. Alternatively a strong T_H_2 response, characterized by an intense but nonprotective humoral reaction, in addition to high IL-4 and IL-10 release, coincides with the lepromatous manifestation. A system of regulation controlling such a delicate balance is certain to be complex, but undoubtedly must begin with pathogen recognition at the sites of host inoculation.

The innate immune system provides an immediate means of surveillance for invading microorganisms and importantly, for a disease such as leprosy, plays a key role in initializing the type of adaptive strategy that the host will use to respond to infection. Pattern recognition receptors (PRRs) are pivotal for innate sensing of conserved microbial-associated molecules indicative of infection in the extracellular milieu. Amongst the PRRs is a class of type-1 integral membrane proteins, the ten-member Toll-like receptor family, whose extracellular domains recognize a diverse array of such microbial agonists. TLRs are expressed by a variety of leukocytes and solid tissue cell types, with the highest levels primarily displayed by cells of myeloid lineage such as monocytes, macrophages, and dendritic cells. However, TLR subsets are also expressed in lymphocytes, epithelium, endothelium, fibroblasts, and even Schwann cells. Upon ligand binding, the TLRs initiate a signal cascade which ultimately activates NF*κ*B-regulated genes, most notably encoding proinflammatory cytokine and chemokines, as well as the costimulatory molecules required for T-cell activation. *M. leprae *and other mycobacterial species such as *M. tuberculosis* are rich in agonists for several members of the TLR family, including TLR1, 2, 4, 6, and 9. 

Genetic association studies have identified a number of single nucleotide polymorphisms (SNPs) in TLR genes which associate with susceptibility or resistance to bacterial and viral infection. Due to the high relevance of host genetic factors in the outcome of *M. leprae* infection, decades of research have been devoted to identifying key polymorphisms which associate strongly with the predisposition to clinical leprosy manifestation, spontaneous clearance, and to the spectrum of disease progression. This paper will examine specific TLR polymorphisms which are associated with *M. leprae* and *M. tuberculosis *infection, with a focus on the potential mechanistic basis for their effects on pathogenesis and host response.

## 2. TLR-Mediated Recognition of Mycobacteria

### 2.1. TLR1/2

The majority of TLRs (TLR3, 4, 5, 7, 8, and 9) signal via homodimerization in the presence of agonist binding. Members of the TLR2 subfamily, which includes TLR1, 2, 6, and 10, are unique in their ability to form heterodimeric complexes which sense an extremely diverse group of microbial molecules. TLR1 heterodimerizes with TLR2 to recognize primarily triacylated lipoproteins displayed by Gram-positive, Gram-negative, and acid-fast bacteria [2–4]. This receptor pair is also capable of detecting glycolipids, glycoproteins, lipopeptides, lipoteichoic acids, and fungal cell wall components [3–8]. Interestingly, the cell wall and membrane of mycobacteria are especially rich in TLR1/2 agonists. The lipoglycans lipomannan (LM) and mannose or arabinose-capped lipoarabinomannan (ManLAM and AraLAM) are major virulence factors in mycobacterial species. ManLAM and AraLAM in particular are potent TLR1/2 agonists and have been shown to contribute both to macrophage activation and immunomodulation of host responses [5, 8]. These molecules behave in large part as scavengers of reactive oxygen species deployed by the phagosomal oxidative burst [9]. In addition, stimulation of macrophages with purified *M. leprae* and *M. tuberculosis* LAM molecules has been shown to decrease IFN*γ*-inducible microbial killing by macrophages, reduce T-cell proliferation and activation, and inhibit protein kinase C, a key transduction molecule in IFN*γ* and respiratory burst signaling [9–11].

Although TLR2 has the ability to dimerize with multiple TLR coreceptors, TLR1/2 heterodimers are the primary sensors by which immune cells recognize mycobacterial lipoproteins [4, 6]. Significant examples of such mycobacterial agonists include the *M. leprae *and *M. tuberculosis* 19 kDa lipoprotein orthologs, ML1966 and LpqH [12]. These membrane-anchored proteins possess adhesin properties and serve as major mycobacterial surface antigens [13]. Like the cell wall lipoglycans, LpqH in particular provides robust immunosuppressive functions. The impressive ability of these triacylated lipoproteins to deactivate macrophages has been well characterized and shown to be dependent on engagement of TLR2 [14–23]. The *M. leprae* annotated genomic sequence predicts 31 different lipoprotein genes, including the enzymatic machinery for ligation of acyl chains [12]. Orthologous partners for all of these genes exist in *M. tuberculosis*, which possesses an additional 60 genes encoding putative lipoproteins. In addition to the well-studied 19 kDa antigen, *M. leprae *also encodes a 33 kda (ML0603) lipoprotein with TLR2 immunostimulatory activity [12]. It is conceivable that many of the triacylated lipoproteins displayed by both leprosy and tuberculosis bacilli could signal via TLR1/2 heterodimers and may possess similarly immunosuppressive functions to those of the 19 kDa antigen. 

Nonlipidated molecules derived from mycobacteria are also capable of binding and signaling through TLR1/2. The 6 kDa early secreted antigenic target (ESAT-6) of *M. tuberculosis* is a highly potent CD4^+^ T-cell antigen and is absent in most nontuberculosis complex mycobacteria [24, 25]. A commonly used diagnostic test for TB, approved by the FDA in 2005, is based upon whole blood stimulation with purified ESAT-6 in an IFN*γ* release assay. Unlike classical proinflammatory TLR agonists, it has been shown that ESAT-6 engages TLR2 in a way that inhibits MyD88-dependent TLR signaling including activation of NF*κ*B and interferon regulatory factors [26]. *M. leprae *possesses an ESAT-6 ortholog with 36% identity, ML0049, and both monocytes and T cells from leprosy patients respond to L-ESAT-6 by secreting IFN*γ* [27]. At this time no direct evidence is available to support an immunosuppressive role for ML0049 similar to that of ESAT-6.

### 2.2. TLR4

While innate immune responses to both *M. leprae *and *M. tuberculosis *rely heavily on TLR1/2 stimulation by various cell wall components, TLR4 has also been shown to play a role in detecting mycobacteria. The classical ligand for TLR4 is lipopolysaccharide (LPS), derived from the outer cell membrane of Gram-negative bacteria. Despite the fact that LPS is absent from mycobacterial membranes, studies in transfected cells and murine macrophages have shown that *M. leprae* and *M. tuberculosis* are both recognized by TLR4 [5, 6]. For *M. tuberculosis*, this effect was linked to MD-2-mediated TLR4 recognition of secreted heat shock protein 65 (HSP-65) and chaperonin 60 protein [28, 29]. Another secreted tuberculosis protein, the adhesin heparin-binding hemagglutinin (HBHA), was found to bind TLR4, induce maturation, and activate proinflammatory cytokine secretion in dendritic cells [30]. *M. leprae* encodes orthologs of HSP60, HSP65, and HBHA, but no studies have examined whether these proteins possess similar TLR4 stimulating qualities to those of *M. tuberculosis*. It has been reported however that LPS binding by TLR4 can be blocked with heat-killed *M. leprae*, inhibiting monocyte secretion of IL-1*β* and IL-6 [31].

### 2.3. TLR9

TLR9 mediates recognition of unmethylated CpG elements in viral and bacterial DNA, a motif relatively rare in vertebrate genomes [32, 33]. Unlike TLR1, 2, and 4, which traffic to the cell surface, TLR9 is predominantly expressed intracellularly in the endoplasmic reticulum [33]. Upon pathogen stimulation, TLR9 localizes to endosomal compartments, where the receptor gains access to genomic DNA released from the degradation of phagocytized bacteria or internalized virus [34]. Primary human monocyte-derived macrophages and murine dendritic cells respond to stimulation with CpG DNA derived from various strains of *M. tuberculosis *(H37Rv and H37Ra) and *M. bovis *(wildtype and BCG) by increasing transcription of TNF*α* and IFN*α* [35, 36]. Interestingly, DNA from the attenuated strains induces a much more vigorous TNF*α* response than DNA from virulent mycobacteria [4].

## 3. TLR Polymorphisms

Given the importance of TLRs in mediating host responses to pathogenic mycobacteria, it is not surprising that single nucleotide polymorphisms effecting expression or function of these receptors influences host susceptibility to leprosy and tuberculosis.

### 3.1. TLR1

A SNP involving a thymine to guanine transversion (T1805G) in TLR1 results in a nonsynonymous substitution at amino acid position 602 (I602S), a position residing in the cytoplasmic region proximal to the receptor's transmembrane domain [37, 38]. Although western blotting and intracellular staining illustrate equivalent protein expression of each variant, both transfection and primary human monocyte studies reveal a trafficking deficiency for TLR1 602S, causing the receptor to be absent from the plasma membrane [38]. In transfected cell lines and monocytes from TLR1 602S/S homozygotes, the subsequent lack of surface TLR1 602S induces a state of hyporesponsiveness to TLR1/2 agonists, including bacterial lipoproteins and synthetic triacylated lipopeptides [37, 38]. These results serve to highlight the importance of TLR localization in receptor function. 

Genetic association studies of leprosy have included DNA samples from diverse populations across the world, including individuals of Eastern Asian, African American, Hispanic, Nepalese, Turkish, and Caucasian backgrounds. Notably, the allelic distribution of TLR1 I602S varies greatly depending on racial ancestry. For example, Caucasian individuals possess the 602I allele at ~25% and the 602S allele at ~75%, while African American individuals possess a reciprocal allele frequency of ~75/25 percent for I/S [38, 39]. Hispanic, Turkish, and Nepalese individuals have I/S ratios of ~70/30, 57/43, and ~94/6 percent, respectively [38–40]. The TLR1 602S allele appears to be virtually absent in East Asian individuals with a 602I allele frequency of >99% [38]. 

Peripheral blood mononuclear cells from TLR1 602S/S individuals were shown to have greatly diminished cytokine production when stimulated with whole irradiated *M. leprae* and *M. tuberculosis*, as well as membrane and cell wall fractions [37–39]. It would therefore be expected that individuals homozygous for TLR1 602S would have increased susceptibility to mycobacterial infection. However, several disease association studies have revealed a protective role for the deficient TLR1 602S variant against the development of both clinical leprosy and tuberculosis [38–41]. The study performed by Johnson et al. observed that a cohort of Turkish leprosy patients had a significantly higher allele frequency for TLR1 602I versus healthy controls. Conversely, the TLR1 602S allele was significantly underrepresented amongst leprosy patients, with an odds ratio for infection of 0.48 (*P* < 0.05) among TLR1 602S/S homozygotes [38]. 

T_H_2-directed lepromatous or borderline forms of leprosy are potentially unstable disease states and may quickly revert to a T_H_1 type adaptive response during a so-called reversal reaction. This can result in a sudden and exacerbated increase in acute T-cell-mediated immunity and create more intense tuberculoid symptoms along with local tissue damage. Misch et al. observed that the TLR1 602S allele confers protection against leprosy reversal reaction (OR = 0.51, *P* = 0.01) [39]. TLR1 I602S also has been shown to influence risk of tuberculosis. Ma et al. observed a significant increase in the rate of extrapulmonary TB infection in African American patients possessing the 602I/I genotype (OR = 2.5, *P* < 0.001) [40]. Striking evidence of this polymorphism's role in leprosy was established when an extensive and unbiased genome-wide array of 2092 genes in 1500 individuals identified TLR1 602S (OR = 0.31, *P* < 0.001) as one of two alleles that afforded the greatest protection against leprosy, the other being the MHCII allele HLA-DRB1/DQA1 (OR = 0.43, *P* < 0.001) [41]. A second polymorphism in TLR1, N248S (A743G), is in strong linkage disequilibrium with the TLR1 602I allele which may explain the finding, observed in a Bangladesh cohort of leprosy patients, that *M. leprae * infection associates with the TLR1 248S/S genotype (OR = 1.34, *P* = 0.02) [37, 39, 42].

### 3.2. TLR2

An insertion/deletion polymorphism in the TLR2 promoter lies at position −196 to −174 bp upstream of the start codon. This polymorphism has been studied in the context of TLR2 expression in both asthma and hepatitis C infection [43, 44]. An *in vitro* reporter construct carrying the deletion allele of the TLR2 promoter induces lower luciferase activity than the insertion allele, suggesting that the former possesses inherently reduced transcriptional activity [44]. In addition, primary human monocytes from individuals carrying the deletion allele produce significantly less IL-8 upon stimulation with peptidoglycan [43]. A subsequent disease association study of Caucasian (OR = 0.41, *P* ≤ 0.001) and African (Guinea-Bissau) (OR = 0.70, *P* = 0.02) tuberculosis cohorts revealed protection via homozygosity for the fully functional insertion allele [45].

A microsatellite marker located between −162 and −100 bp in the promoter region of TLR2 contains two adjacent variable number tandem repeats of CT and TG which vary in length between 280–290 bp [46, 47]. An investigation of Ethiopian leprosy patients by Bochud et al. revealed a lower frequency of the 290bp repeat allele in disease cases versus healthy controls (OR = 0.62, *P* = 0.02) [46]. In addition, a 288 bp allele was observed less frequently in lepromatous versus tuberculoid leprosy patients (OR = 0.49, *P* = 0.02). However, this same allele was also shown to greatly increase susceptibility to reversal reaction (OR = 5.83, *P* = 0.001) in a subgroup of patients which had been followed for 8 additional years [46]. Using an *in vitro* reporter assay, Yim et al. revealed that variability in the number of microsatellite repeats effected TLR2 promoter function in response to cytokine stimulation [47].

No phenotypic effects on TLR2 function have been linked to a synonymous SNP (N199N, C597T) in the extracellular domain of this receptor. However, the 8-year leprosy reaction followed up by Bochud et al. identified a protective role for TLR2 597T against reversal reaction (OR = 0.34, *P* = 0.002) [46]. This polymorphism was also shown to be highly relevant in a Vietnamese cohort of TB patients where progression of disease was more than twofold higher and dissemination of bacilli to the brain was 3-fold higher in individuals homozygous for the 597C allele [48].

The G2258A SNP (R753Q) in the cytoplasmic domain of TLR2 has been well characterized as a functionally deficient variant with reduced responses to bacterial lipoproteins and synthetic di- and triacylated lipopeptides [49, 50]. Most reports on TLR2 R753Q have shown inhibition of signaling in response to *Borrelia burgdorferi* lipoproteins, such as OspA and whole cell lysates. One report observed a lower frequency of TLR2 753Q in Lyme disease patients than healthy controls (OR = 0.39, *P* = 0.033) and an even stronger protective effect was conferred in late stage Lyme disease patients (OR = 0.16, *P* = 0.018) [50]. Interestingly, the converse result was obtained when a Turkish cohort of tuberculosis patients was genotyped for the TLR2 polymorphism. In this study, the risk of developing tuberculosis was increased over 6-fold for TLR2753Q/Q individuals and 1.6-fold for heterozygotes [51]. Another study of Turkish pediatric patients also associated the TLR2 R753Q heterozygotes with TB, citing an over 5-fold increased risk of infection (OR = 5.05, *P* ≤ 0.001) [52]. Taken together these results indicate that a functionally deficient TLR2 variant plays opposite roles in two different infectious diseases: protection in the context of Lyme disease and susceptibility in the context of TB. Although no disease association studies have been performed between TLR2 R753Q and leprosy, it would be very interesting to see if the results observed in the context of TB would also extend to *M. leprae*.

### 3.3. TLR4

Numerous studies of two TLR4 polymorphisms, D299G (G896A) and T399I (C1196T), have revealed increased risk of infection with several Gram-negative organisms as well as septic shock [53–55]. These SNPs, which alter amino acids in the extracellular domain of TLR4, appear to affect receptor function depending upon the experimental system under investigation. Some studies have reported a decrease in LPS-induced IL-12 and IL-10 in asthma patients, reduced cytokine secretion in an inhaled LPS human model, and inhibited signaling in cell-based transfection models [56–58]. However, other studies have found no functional deficits exhibited by primary human monocytes and PBMCs obtained from individuals who are either heterozygous or homozygous for these TLR4 variants [59–61].

Similar to TLR2, deficient TLR4 function is generally associated with increased susceptibility to mycobacterium infection. For example, a significant increase in the frequency of the TLR4 299G allele was observed in pulmonary tuberculosis patients in an Asian Indian cohort (OR = 2.1, *P* = 0.001), with highest bacillary loads observed in homozygous individuals [62]. Other studies in HIV/TB coinfected patients indicate that 299G is a risk factor for active tuberculosis in Mediterranean Caucasians (OR = 2.0) and Tanzanian patients (OR = 2.8, *P* = 0.06), but at borderline statistical significance [63, 64]. No statistically significant findings were associated with TLR4 T399I for TB [62–64]. In contrast to *M. tuberculosis*, a protective role for TLR4 D299G has been identified in association with *M. leprosy *infection. Bochud et al. examined an Ethiopian population and observed lower frequencies of TLR4 D299G (OR = 0.34, *P* < 0.001) and T399I (OR = 0.16, *P* < 0.001) among leprosy patients [31]. Conversely, another investigation found TLR4 D299G in a Malawi cohort of leprosy patients afforded no protection, although their cohort was half the size of the previous study [64].

### 3.4. TLR9

Velez et al. investigated the prevalence of several TLR9 polymorphisms in tuberculosis patients of Caucasian, African American, and African (Guinea-Bissau) descent [45]. No functional studies have been performed examining the effects of these polymorphisms on TLR9 signaling. However, two of these gene-flanking polymorphisms, rs352139 and rs5743836, appear to confer protection to TB. rs352139 was observed less frequently in Caucasian (OR = 0.53, *P* = 0.017) and African American (OR = 0.58, *P* = 0.029) TB patients, while rs5743836 provided protection in African Americans (OR = 0.54, *P* = 0.024) and in Caucasians (OR = 0.50, *P* = 0.015) [45]. No statistically significant reduction in risk was associated with members of the Guinea-Bissau cohort.

## 4. Subversion of TLR Signaling by Mycobacteria

Based upon the established role of TLRs in host defense, polymorphisms which negatively affect receptor function would be expected to increase susceptibility and worsen outcomes to infection. This generality holds true for a number of TLR polymorphisms in various infectious disease settings and simply reflects the host's inability to properly recognize and respond to bacterial agonists. However, a number of functionally deficient polymorphisms in TLR1 and TLR4 have been found to confer protection to mycobacterial infection. This finding suggests that, during the course of evolution, mycobacteria have subverted the TLR system in ways that are advantageous to establishing and maintaining infection. Much of the experimental evidence supporting this idea is provided below.

### 4.1. Adaptive Response

**Figure 1:**
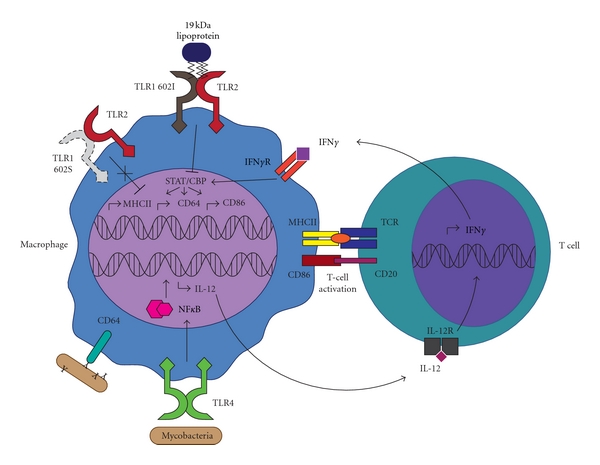
Stimulation of macrophage TLRs by bacterial products activates NF*κ*B which drives transcription of interleukin-12, along with other cytokines and chemokines that subsequently recruit and instruct T cells. Secreted IL-12 stimulates T cells to produce IFN*γ*, which serves to further stimulate the macrophage. Activation of macrophages by IFN*γ* leads to induction of transcription factors such as STAT1 and CBP, which facilitate production of transactivators, including CIITA. This results in the transcription of multiple IFN*γ*-regulated genes, including MHCII, CD86, and CD64. MHCII is required for antigen presentation and, along with CD86, is used by macrophages to activate CD4^+^ T-helper cells. Antibody-opsonized particles are recognized by the phagocytic receptor, CD64, which binds the Fc portion of IgG. TLR1 602I traffics to the cell surface where it forms heterodimers with TLR2 to sense microbial agonists. Binding of the mycobacterial 19 kDa lipoprotein to TLR1/2 has been shown to block STAT1-CBP activity, thereby dampening the induction of IFN*γ*-regulated genes. It is possible that the trafficking-deficient TLR1 602S allele (dashed lines) confers protection against leprosy and TB by preventing the subversion of IFN*γ* signaling by these mycobacterial agonists.

Several studies have revealed that prolonged mycobacterial stimulation via TLR1/2 causes macrophages to become refractory to IFN*γ* (Figure 1) [14–23]. IFN*γ*, a type-2 interferon primarily secreted by T cells and natural killer cells, is a T_H_1-skewing cytokine essential to the containment of mycobacterial infection. This secreted dimeric glycoprotein is a classical activator of innate immune effectors and plays a key role in the induction of macrophage microbicidal functions, including phagolysosome maturation and oxidative burst. In addition, IFN*γ* facilitates a priming function by upregulating the cellular machinery required for antigen presentation, phagocytosis, and T-cell costimulation. Mice deficient in cytokine production or the IFN*γ* receptor develop disseminating and ultimately fatal mycobacterial disease [65]. Also, a human mutation linked to IFN*γ* signaling associates with susceptibility mycobacterial disease and disseminated infection following vaccination with the Calmette-Guèrin bacillus strain of *M. tuberculosis* [66]. 

TLR activation plays an important role in IFN*γ* production by T cells. Active NF*κ*B provided by TLR ligation on macrophages or dendritic cells induces expression and secretion of IL-12, a cytokine essential to the T_H_1 skewing of CD4^+^ T-helper cells. Once exposed to IL-12, these cells begin secreting IFN*γ*, establishing a positive IFN*γ*/IL-12 feedback loop (Figure 1). However, stimulation with the *M. tuberculosis* 19 kDa lipoprotein is sufficient to greatly reduce induction of IFN*γ*-regulated genes in many cell types, including murine RAW264.7 cells, mouse bone-marrow-derived macrophages, human THP-1 cells, and primary human monocytes (Figure 1). Microarray analysis has shown an inhibition of IFN*γ*-inducible transcription of IL-12 receptor mRNA upon stimulation with mycobacterial lipoproteins. This provides direct interference with the IFN*γ*-IL-12 signaling axis. Further subversion of TLR1/2 signaling is illustrated by abrogated IFN*γ*-dependent upregulation of macrophage activation markers MHCII, CD64, and CD86 in cells stimulated with either whole mycobacteria or purified triacylated lipoproteins. MHCII is a receptor essential for the activation of adaptive immunity by professional antigen-presenting cells (APCs). Forced downregulation of MHCII by mycobacteria corresponds to reduced antigen processing in murine bone-marrow-derived macrophages and associates with a curtailed ability to activate T cells. Phagocyte-expressed CD86 (B7.2) provides costimulatory signals which, in addition to MHCII, are essential for CD4^+^ T-helper-cell activation and survival. Simultaneous inhibition of CD86 and MHCII by TLR1/2 activation would impart a major hindrance on recruitment of adaptive help at the site of infection. Finally, CD64 (Fc*γ*RI), a key phagocytic receptor specific for IgG-opsonized pathogens, facilitates pathogen internalization and induction of the oxidative burst. Downregulation of this receptor could potentially reduce phagocytic uptake of mycobacteria by macrophages, dendritic cells, and neutrophils, thereby decreasing pathogen clearance and generation of peptides for antigen presentation.

*M. tuberculosis* also appears to subvert host adaptive immunity through secretion of ESAT-6. The engagement of TLR2 by ESAT-6 inhibits IFN*γ* induction of CTIIA, the transcriptional coactivator required for MHCII expression [67]. Furthermore, the TLR2-dependent inhibition of IFN*γ* signaling by mycobacterial lipoproteins was shown to act through a similar mechanism, whereby inhibition of CIITA blocks induction of many macrophage activation markers [21, 22, 68].

### 4.2. Direct Killing of Mycobacteria

**Figure 2:**
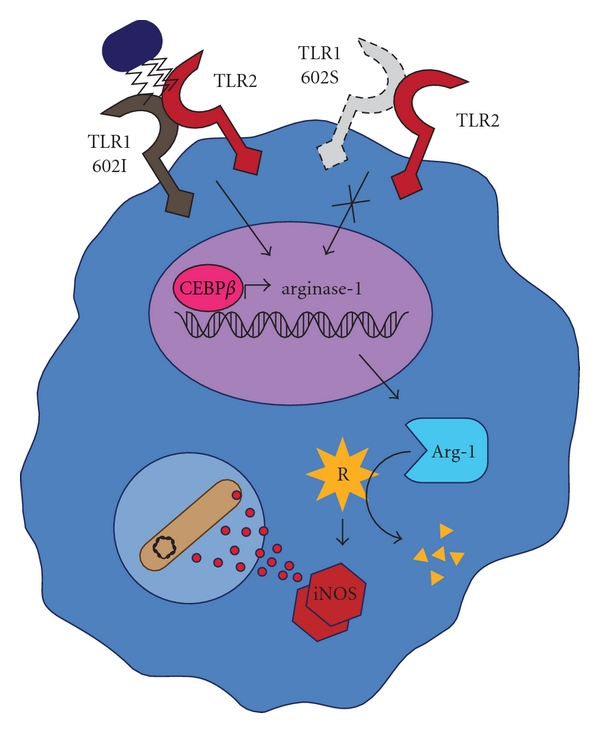
Arginine (R) is required as a substrate for inducible nitric oxide synthase (iNOS) in the production of nitric oxide. Nitric oxide contributes to microbial killing by combining with reactive oxygen intermediates in the phagolysosome to produce highly toxic peroxynitrite. Activation of surface TLR2 and TLR1 602I by mycobacterial products, including the 19 kDa lipoprotein, induces transcription of arginase-1 in a CEBP*β*-dependent manner. Arginase-1 reduces substrate availability for iNOS by breaking down arginine, reducing the production of reactive oxygen species and favoring mycobacterial survival. TLR1 602S does not traffic to the cell surface (dashed lines) and therefore does not form a TLR1/2 signaling complex which would normally dampen the oxidative burst.

Another recent TLR2-dependent immunoregulatory mechanism used by mycobacteria has been identified which affects the macrophage respiratory burst [70]. Inducible nitric oxide synthase (iNOS) generates nitric oxide (NO), a major component of the phagolysosomal oxidative burst. NO reacts with superoxide, a product of NADPH oxidase, to generate peroxynitrite, a powerful oxidant capable of damaging diverse biological molecules within phagocytized pathogens. iNOS requires cellular pools of L-arginine to drive catalysis and the availability of this substrate has been shown to be a rate-limiting step in production of NO. Arginase-1, a metabolic enzyme important in the urea cycle, shares arginine as a substrate. This enzyme participates in the final step of the cycle by metabolizing L-arginine to L-ornithine and urea. Curiously, it has been reported that during *Mycobacterium bovis *(BCG) infection of J774.1 mouse macrophages, the levels of urea rise with increasing bacterial replication [71]. A recent study has linked these observations to mycobacterial pathogenesis by showing that stimulation of primary mouse macrophages with BCG upregulates levels of both arginase-1 mRNA and protein [72]. Surprisingly, TLR2 and MyD88 knockout mice resist upregulation of arginase-1 [72]. In addition, Arginase-1 knockouts exhibit enhanced NO production in BCG-infected macrophages and lower bacterial burdens in an aerosol infection model of *M. tuberculosis *[72]. Another report found that cell supernatants from BCG-infected macrophages are capable of inducing arginase-1 in uninfected neighboring macrophages, an effect dependent on the production of IL-6 and IL-10 [73]. These data suggest that mycobacterial stimulation of TLR2 induces arginase-1, which inhibits the oxidative burst and allows increased survival within the macrophage endosome (Figure 2). Similar to the inhibition of IFN*γ* signaling, Arginase-1 mediated immunosuppression was found to be dependent on CEBP-*β* [72].

### 4.3. Nerve Damage

**Figure 3:**
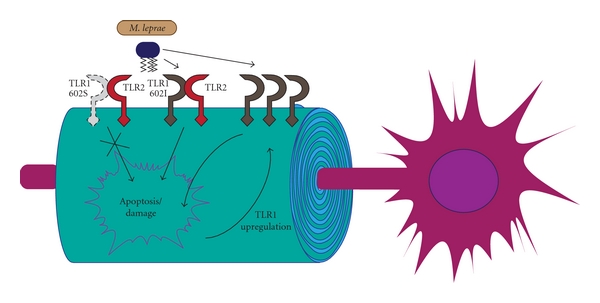
Schwann cells displaying TLR1 602I and TLR2 are stimulated by the *M. leprae* 19 kDa lipoprotein to commence programmed cell death. Damage to peripheral nerves, by either direct infection or inflammatory ischemia, induces TLR1 expression which, in combination with TLR2 and mycobacterial products, can amplify apoptotic signals. Upregulation of TLR1 from damage caused by either direct infection or inflammatory ischemia could predispose nerves to further injury, resulting in classical leprosy anesthesis. Cells expressing the trafficking-deficient TLR1 602S may resist this mechanism of nerve damage.

Colonization of Schwann cells by *M. leprae* is known to stimulate granuloma formation and cell-mediated nerve injury [74]. The influx of numerous adaptive and innate immune cells proximal to an infected peripheral nerve may produce direct Schwann cell damage and killing, or indirect death via pressure-induced ischemia. Immunohistochemistry of skin biopsies from both lepromatous and tuberculoid leprosy patients reveals TLR1 and TLR2 surface expression by multiple cell types, including Schwann cells [12, 75]. Schwann cell TLR2 is functional as indicated by the activation of NF*κ*B by synthetic bacterial lipopeptides [75, 76]. TLR2-dependent activation by the 19 kDa lipoprotein of *M. leprae *has also been shown to induce apoptosis in primary human Schwann cells. This effect was reduced when cells were incubated with blocking anti-TLR2 antibodies [75]. Additionally, nerve injury promotes massive upregulation of TLR1, manyfold over most other Schwann cell-expressed TLRs [76]. Since *M. leprae *is capable of activating apoptosis in a TLR2, and presumably TLR1-dependent manner, it is possible that upregulation of TLR1 by nerve damage may establish a positive feedback loop for Schwann cell deterioration (Figure 3). Apoptotic properties have also been noted for the *M. tuberculosis* 19 kDa antigen. López et al. revealed a TLR2-dependent induction of apoptosis in monocyte-derived macrophages stimulated with the triacylated lipoprotein [77]. 

## 5. SNP Protection against Mycobacterial Infection

Collectively, the above studies convincingly highlight the role of TLR1 and TLR2 in mycobacterial pathogenesis. Based on the established immunosuppressive activity of the TLR1/2 complex following recognition of mycobacterial agonists, it is not difficult to imagine why a functionally deficient allele of TLR1 would be advantageous. If TLR1 602S is unable to gain access to the cell surface, where it is proximal both to its coreceptor and bacterial ligand, immune evasion through the TLR1/2 signaling complex would not be achieved by mycobacteria (Figures 1, 2, and 3). One could take this a step further to suggest that TLR1 602S/S homozygous individuals infected with *M. leprae* are more likely to develop the less severe tuberculoid form of the disease. It is intriguing to note that similar to TLR1, but unlike TLR2, deficient TLR4 functions are generally associated with increased resistance to *M. leprae*. If the D299G and T399I SNPs do indeed confer hyporesponsiveness to TLR4 agonists, the protection to mycobacteria infection is analogous to the trafficking-deficient TLR1 I602S. At this time no groups have examined the potential for mycobacteria-derived TLR4 agonists to induce immunosuppressive effects similar to those of TLR1/2 ligands. Such studies could provide a mechanism by which deficient TLR4 signaling provides resistance to mycobacteria.

## 6. Conclusions

Mycobacteria have been significant human pathogens for thousands of years. The earliest known human case of leprosy has been traced back to over 4000 years ago from East Indian skeletal remains [78]. Human infection by *M. tuberculosis* is arguably even more ancient, as evidenced by 9000-year-old bone samples from Neolithic settlements testing positive for TB DNA [79]. The extreme success for a pathogenic organism such as mycobacteria requires either high rates of transmission or powerful immunoevasive strategies. Transmissibility for tuberculosis is not particularly profound and only occurs during relatively rare active phases. The likelihood of passing leprosy is equally low; yet these pathogens are consistently endemic worldwide. Over many decades, the exquisite mechanisms by which mycobacteria neutralize and even subvert host defenses have been uncovered, potentially explaining the impressive effectiveness of these pathogens.

 Highlighted in this paper are the TLR-mediated pathways by which *M. leprae *and *M. tuberculosis* are recognized by the innate immune system. Through many functional and genetic studies it can be concluded that single nucleotide polymorphisms in TLRs have the ability to both modify receptor function and associate with risk of leprosy and tuberculosis (Table 1). Some investigations have identified SNPs which negatively affect TLR signaling and correspondingly induce hyporesponsiveness in immune cells to these bacteria. In some cases, such as TLR2 in the context of TB and leprosy and TLR4 in the context of TB, this abrogated function correlates unsurprisingly with increased susceptibility of the host to infection. Unexpectedly, however, this trend is not universal. The I602S polymorphism of TLR1 inhibits trafficking of the receptor to the cell surface thereby abrogating innate recognition of mycobacterial agonists. This functional deficiency in TLR1 is not associated with an increased risk of infection but instead reduces the likelihood of developing clinical leprosy by over half. In addition, SNPs which appear to impair TLR4 function (D299G, T399I) reduce the incidence of leprosy infection by over two-thirds.

Many of the known immunoevasive strategies employed by mycobacteria operate through TLR activation by bacterial components. It is intriguing to imagine that mycobacteria have evolved over the centuries to subvert host responses in a way that have aided them in their obvious success as human pathogens. By creating functionally poor responses to TLR agonists, these SNPs have effectively counteracted such a strategy. Indeed, the TLR1-6-10 gene cluster has been identified as a hotspot of strong positive selection [80, 81]. It is of interest to note that the geographic distribution of the TLR1 602I allele, which increases susceptibility to *M. leprae* by 1.5 times, is highest in regions where leprosy is most endemic. Such genetic diversity in TLRs is certain to provide further important insights into both pathogenesis and host defense against mycobacteria.

## Figures and Tables

**Table 1:** TLR polymorphisms are organized by receptor, genotype, phenotype, and cohort. The odds ratio (OR) measures the strength of association between genotype and disease. A ratio less than 1 depicts the degree of protection and a ratio greater than 1 depicts the factor of increased susceptibility. Reference numbers are included with the associated cohort. RR: reversal reaction; del: deletion; ins: insertion; VNTR: variable number tandem repeat.

Gene	SNP		Reference Sequence	OR		*P* value	Cohort
	Amino acid	DNA		*M. leprae*	*M. tuberculosis*		
*TLR1*	I602S	A1805G	rs5743618	0.48		<0.005	Turkish [38]
				0.51 (RR)		0.01	Nepalese [39]
				0.31		<0.001	Asian Indian [41]
		A/A			2.5 (extrapulmonary)	<0.001	African American [40]
	N248S	A743G	rs4833095	1.24		0.02	Bangladesh [42]
*TLR2*	~	del (−196 to −174)ins			0.70	0.02	Guinea-Bissau [45]
		I/I			0.41	<0.001	Caucasian [45]
	~	VNTR −162 to −100					
		290 bp		0.62		0.02	Ethiopian [46]
		288 bp		5.83 (RR)		0.001	Ethiopian [46]
				0.49 (lepromatous)		0.02	Ethiopian [46]
	N199N	C597T	rs3804099	0.34 (RR)		0.002	Ethiopian [46]
		C/C			2.22	0.007	Vietnamese [48]
					3.33 (meningitis)	0.004	Vietnamese [48]
	R753Q	G2258A (A/A)	rs5743708		6.04	0.022	Turkish [51]
		A/G			1.60	0.59 (*χ* ^2^)	Turkish [51]
					5.05	<0.001	Turkish pediatric [52]
*TLR4*	D299G	G896A	rs4986790	0.34		<0.001	Ethiopian [31]
					2.10	<0.001	Asian Indian [62]
					2.00	~	Mediterranean Caucasian [69]
					2.80	0.06	Tanzanian [63]
	T399I	C1196T	rs4986791	0.16		<0.001	Ethiopian [31]
*TLR9*	~	G-A	rs352143		0.58	0.029	African American [45]
					0.53	0.017	Caucasian [45]
	~	C-T	rs5743836		0.54	0.024	African American [45]
					0.50	0.015	Caucasian [45]

## References

[1] Montoya D, Modlin RL (2010). Learning from Leprosy. Insight into the human innate immune response. *Advances in Immunology*.

[2] Aliprantis AO, Yang RB, Mark MR (1999). Cell activation and apoptosis by bacterial lipoproteins through Toll-like receptor-2. *Science*.

[3] Yoshimura A, Lien E, Ingalls RR, Tuomanen E, Dziarski R, Golenbock D (1999). Cutting edge: recognition of Gram-positive bacterial cell wall components by the innate immune system occurs via Toll-like receptor 2. *Journal of Immunology*.

[4] Takeuchi O, Sato S, Horiuchi T (2002). Cutting edge: role of Toll-like receptor 1 in mediating immune response to microbial lipoproteins. *Journal of Immunology*.

[5] Means TK, Lien E, Yoshimura A, Wang S, Golenbock DT, Fenton MJ (1999). The CD14 ligands lipoarabinomannan and lipopolysaccharide differ in their requirement for toll-like receptors. *Journal of Immunology*.

[6] Means TK, Wang S, Lien E, Yoshimura A, Golenbock DT, Fenton MJ (1999). Human Toll-like receptors mediate cellular activation by *Mycobacterium tuberculosis*. *Journal of Immunology*.

[7] Schwandner R, Dziarski R, Wesche H, Rothe M, Kirschning CJ (1999). Peptidoglycan- and lipoteichoic acid-induced cell activation is mediated by Toll-like receptor 2. *Journal of Biological Chemistry*.

[8] Tapping RI, Tobias PS (2003). Mycobacterial lipoarabinomannan mediates physical interactions between TLR1 and TLR2 to induce signaling. *Journal of Endotoxin Research*.

[9] Chan J, Fan X, Hunter SW, Brennan PJ, Bloom BR (1991). Lipoarabinomannan, a possible virulence factor involved in persistence of *Mycobacterium tuberculosis* within macrophages. *Infection and Immunity*.

[10] Kaplan G, Gandhi RR, Weinstein DE (1987). Mycobacterium leprae antigen-induced suppression of T cell proliferation in vitro. *Journal of Immunology*.

[11] Sibley LD, Hunter SW, Brennan PJ, Krahenbuhl JL (1988). Mycobacterial lipoarabinomannan inhibits gamma interferon-mediated activation of macrophages. *Infection and Immunity*.

[12] Krutzik SR, Ochoa MT, Sieling PA (2003). Activation and regulation of Toll-like receptors 2 and 1 in human leprosy. *Nature Medicine*.

[13] Diaz-Silvestre H, Espinosa-Cueto P, Sanchez-Gonzalez A (2005). The 19-kDa antigen of *Mycobacterium tuberculosis* is a major adhesin that binds the mannose receptor of THP-1 monocytic cells and promotes phagocytosis of mycobacteria. *Microbial Pathogenesis*.

[14] Banaiee N, Kincaid EZ, Buchwald U, Jacobs WR, Ernst JD (2006). Potent inhibition of macrophage responses to IFN-*γ* by live virulent *Mycobacterium tuberculosis* is independent of mature mycobacterial lipoproteins but dependent on TLR2. *Journal of Immunology*.

[15] Drage MG, Pecora ND, Hise AG (2009). TLR2 and its co-receptors determine responses of macrophages and dendritic cells to lipoproteins of *Mycobacterium tuberculosis*. *Cellular Immunology*.

[16] Fortune SM, Solache A, Jaeger A (2004). *Mycobacterium tuberculosis* inhibits macrophage responses to IFN-*γ* through myeloid differentiation factor 88-dependent and -independent mechanisms. *Journal of Immunology*.

[17] Gehring AJ, Rojas RE, Canaday DH, Lakey DL, Harding CV, Boom WH (2003). The *Mycobacterium tuberculosis* 19-kilodalton lipoprotein inhibits gamma interferon-regulated HLA-DR and Fc*γ*R1 on human macrophages through toll-like receptor 2. *Infection and Immunity*.

[18] Lafuse WP, Alvarez GR, Curry HM, Zwilling BS (2006). *Mycobacterium tuberculosis* and Mycobacterium avium inhibit IFN-*γ*-induced gene expression by TLR2-dependent and independent pathways. *Journal of Interferon and Cytokine Research*.

[19] Mahon RN, Rojas RE, Fulton SA, Franko JL, Harding CV, Boom WH (2009). *Mycobacterium tuberculosis* cell wall glycolipids directly inhibit CD4 + T-cell activation by interfering with proximal T-cell-receptor signaling. *Infection and Immunity*.

[20] Noss EH, Pai RK, Sellati TJ (2001). Toll-like receptor 2-dependent inhibition of macrophage class II MHC expression and antigen processing by 19-kDa lipoprotein of *Mycobacterium tuberculosis*. *Journal of Immunology*.

[21] Pai RK, Convery M, Hamilton TA, Henry Boom W, Harding CV (2003). Inhibition of IFN-*γ*-induced class II transactivator expression by a 19-kDa lipoprotein from *Mycobacterium tuberculosis*: a potential mechanism for immune evasion. *Journal of Immunology*.

[22] Pennini ME, Pai RK, Schultz DC, Boom WH, Harding CV (2006). *Mycobacterium tuberculosis* 19-kDa lipoprotein inhibits IFN-*γ*-induced chromatin remodeling of MHC2TA by TLR2 and MAPK signaling. *Journal of Immunology*.

[23] Pai RK, Pennini ME, Tobian AAR, Canaday DH, Boom WH, Harding CV (2004). Prolonged toll-like receptor signaling by *Mycobacterium tuberculosis* and its 19-kilodalton lipoprotein inhibits gamma interferon-induced regulation of selected genes in macrophages. *Infection and Immunity*.

[24] Harboe M, Oettinger T, Wiker HG, Rosenkrands I, Andersen P (1996). Evidence for occurrence of the ESAT-6 protein in *Mycobacterium tuberculosis* and virulent *Mycobacterium bovis* and for its absence in Mycobacterium bovis BCG. *Infection and Immunity*.

[25] Sorensen AL, Nagai S, Houen G, Andersen P, Andersen AB (1995). Purification and characterization of a low-molecular-mass T-cell antigen secreted by *Mycobacterium tuberculosis*. *Infection and Immunity*.

[26] Pathak SK, Basu S, Basu KK (2007). Direct extracellular interaction between the early secreted antigen ESAT-6 of *Mycobacterium tuberculosis* and TLR2 inhibits TLR signaling in macrophages. *Nature Immunology*.

[27] Geluk A, Van Meijgaarden KE, Franken KLMC (2002). Identification and characterization of the ESAT-6 homologue of Mycobacterium leprae and T-cell cross-reactivity with *Mycobacterium tuberculosis*. *Infection and Immunity*.

[28] Bulut Y, Michelsen KS, Hayrapetian L (2005). *Mycobacterium tuberculosis* heat shock proteins use diverse toll-like receptor pathways to activate pro-inflammatory signals. *Journal of Biological Chemistry*.

[29] Cehovin A, Coates ARM, Hu Y (2010). Comparison of the moonlighting actions of the two highly homologous chaperonin 60 proteins of *Mycobacterium tuberculosis*. *Infection and Immunity*.

[30] Jung D, Jeong SK, Lee C-M (2011). Enhanced efficacy of therapeutic cancer vaccines produced by Co-treatment with mycobacterium tuberculosis heparin-binding hemagglutinin, a novel TLR4 agonist. *Cancer Research*.

[31] Bochud PY, Sinsimer D, Aderem A (2009). Polymorphisms in toll-like receptor 4 (TLR4) are associated with protection against leprosy. *European Journal of Clinical Microbiology and Infectious Diseases*.

[32] Chuang TH, Ulevitch RJ (2000). Cloning and characterization of a sub-family of human Toll-like receptors: hTLR7, hTLR8 and hTLR9. *European Cytokine Network*.

[33] Leifer CA, Kennedy MN, Mazzoni A, Lee C, Kruhlak MJ, Segal DM (2004). TLR9 is localized in the endoplasmic reticulum prior to stimulation. *Journal of Immunology*.

[34] Ahmad-Nejad P, Häcker H, Rutz M, Bauer S, Vabulas RM, Wagner H (2002). Bacterial CpG-DNA and lipopolysaccharides activate toll-like receptors at distinct cellular compartments. *European Journal of Immunology*.

[35] Kiemer AK, Senaratne RH, Hoppstädter J (2008). Attenuated activation of macrophage TLR9 by DNA from virulent mycobacteria. *Journal of Innate Immunity*.

[36] Bafica A, Scanga CA, Feng CG, Leifer C, Cheever A, Sher A (2005). TLR9 regulates Th1 responses and cooperates with TLR2 in mediating optimal resistance to *Mycobacterium tuberculosis*. *Journal of Experimental Medicine*.

[37] Hawn TR, Misch EA, Dunstan SJ (2007). A common human TLR1 polymorphism regulates the innate immune response to lipopeptides. *European Journal of Immunology*.

[38] Johnson CM, Lyle EA, Omueti KO (2007). Cutting edge: a common polymorphism impairs cell surface trafficking and functional responses of TLR1 but protects against leprosy. *Journal of Immunology*.

[39] Misch EA, Macdonald M, Ranjit C (2008). Human TLR1 deficiency is associated with impaired mycobacterial signaling and protection from leprosy reversal reaction. *PLoS Neglected Tropical Diseases*.

[40] Ma X, Liu Y, Gowen BB, Graviss EA, Clark AG, Musser JM (2007). Full-exon resequencing reveals toll-like receptor variants contribute to human susceptibility to tuberculosis disease. *PLoS ONE*.

[41] Wong SH, Gochhait S, Malhotra D (2010). Leprosy and the adaptation of human toll-like receptor 1. *PLoS Pathogens*.

[42] Schilling RP, Hamann L, Faber WR (2009). Polymorphism N248S in the human Toll-like receptor 1 gene is related to leprosy and leprosy reactions. *Journal of Infectious Diseases*.

[43] Nischalke H-D, Coenen M, Berger C (2011). The toll-like receptor 2 (TLR2)-196 to-174 del/ins polymorphism affects viral loads and susceptibility to hepatocellular carcinoma in chronic hepatitis C. *International Journal of Cancer*.

[44] Noguchi E, Nishimura F, Fukai H (2004). An association study of asthma and total serum immunoglobin E levels for Toll-like receptor polymorphisms in a Japanese population. *Clinical and Experimental Allergy*.

[45] Velez DR, Wejse C, Stryjewski ME (2010). Variants in toll-like receptors 2 and 9 influence susceptibility to pulmonary tuberculosis in Caucasians, African-Americans, and West Africans. *Human Genetics*.

[46] Bochud PY, Hawn TR, Siddiqui MR (2008). Toll-like receptor 2 (TLR2) polymorphisms are associated with reversal reaction in leprosy. *Journal of Infectious Diseases*.

[47] Yim J-J, Ding L, Schäffer AA, Park GY, Shim Y-S, Holland SM (2004). A microsatellite polymorphism in intron 2 of human Toll-like receptor 2 gene: functional implications and racial differences. *FEMS Immunology and Medical Microbiology*.

[48] Thuong NTT, Hawn TR, Thwaites GE (2007). A polymorphism in human TLR2 is associated with increased susceptibility to tuberculous meningitis. *Genes and Immunity*.

[49] Lorenz E, Mira JP, Cornish KL, Arbour NC, Schwartz DA (2000). A novel polymorphism in the toll-like receptor 2 gene and its potential association with staphylococcal infection. *Infection and Immunity*.

[50] Schröder NWJ, Diterich I, Zinke A (2005). Heterozygous Arg753Gln polymorphism of human TLR-2 impairs immune activation by Borrelia burgdorferi and protects from late stage lyme disease. *Journal of Immunology*.

[51] Ogus AC, Yoldas B, Ozdemir T (2004). The Arg753Gln polymorphism of the human Toll-like receptor 2 gene in tuberculosis disease. *European Respiratory Journal*.

[52] Dalgic N, Tekin D, Kayaalti Z (2011). Arg753Gln polymorphism of the human Toll-like receptor 2 gene from infection to disease in pediatric tuberculosis. *Human Immunology*.

[53] Agnese DM, Calvano JE, Hahm SJ (2002). Human toll-like receptor 4 mutations but not CD14 polymorphisms are associated with an increased risk of gram-negative infections. *Journal of Infectious Diseases*.

[54] Hawn TR, Verbon A, Janer M, Zhao LP, Beutler B, Aderem A (2005). Toll-like receptor 4 polymorphisms are associated with resistance to Legionnaires’ disease. *Proceedings of the National Academy of Sciences of the United States of America*.

[55] Lorenz E, Mira JP, Frees KL, Schwartz DA (2002). Relevance of mutations in the TLR4 receptor in patients with gram-negative septic shock. *Archives of Internal Medicine*.

[56] Fagerås Böttcher M, Hmani-Aifa M, Lindström A (2004). A TLR_4_ polymorphism is associated with asthma and reduced lipopolysaccharide-induced interleukin-12(p70) responses in Swedish children. *Journal of Allergy and Clinical Immunology*.

[57] Schmitt C, Humeny A, Becker CM, Brune K, Pahl A (2002). Polymorphisms of TLR4: rapid genotyping and reduced response to lipopolysaccharide of TLR4 mutant alleles. *Clinical Chemistry*.

[58] Schwartz DA (2001). The role of TLR4 in endotoxin responsiveness in humans. *Journal of Endotoxin Research*.

[59] Douville RN, Lissitsyn Y, Hirschfeld AF (2010). TLR4 Asp299Gly and Thr399ile polymorphisms: no impact on human immune responsiveness to LPS or respiratory syncytial virus. *PLoS ONE*.

[60] Erridge C, Stewart J, Poxton IR (2003). Monocytes heterozygous for the Asp299Gly and Thr399Ile mutations in the toll-like receptor 4 gene show no deficit in lipopolysaccharide signalling. *Journal of Experimental Medicine*.

[61] Van Der Graaf C, Kullberg BJ, Joosten L (2005). Functional consequences of the Asp299Gly Toll-like receptor-4 polymorphism. *Cytokine*.

[62] Najmi N, Kaur G, Sharma SK, Mehra NK (2010). Human Toll-like receptor 4 polymorphisms TLR4 Asp299Gly and Thr399Ile influence susceptibility and severity of pulmonary tuberculosis in the Asian Indian population. *Tissue Antigens*.

[63] Ferwerda B, Kibiki GS, Netea MG, Dolmans WMV, Van Der Ven AJ (2007). The toll-like receptor 4 Asp299Gly variant and tuberculosis susceptibility in HIV-infected patients in Tanzania. *AIDS*.

[64] Fitness J, Floyd S, Warndorff DK (2004). Large-scale candidate gene study of leprosy susceptibility in the Karonga district of northern Malawi. *American Journal of Tropical Medicine and Hygiene*.

[65] Cooper AM, Dalton DK, Stewart TA, Griffin JP, Russell DG, Orme IM (1993). Disseminated tuberculosis in interferon *γ* gene-disrupted mice. *Journal of Experimental Medicine*.

[66] Cunningham JA, Kellner JD, Bridge PJ, Trevenen CL, McLeod DR, Davies HD (2000). Disseminated bacille Calmette-Guerin infection in an infant with a novel deletion in the interferon-gamma receptor gene. *International Journal of Tuberculosis and Lung Disease*.

[67] Kumar P, Agarwal R, Siddiqui I, Vora H, Das G, Sharma P ESAT6 differentially inhibits IFN-*γ*-inducible class II transactivator isoforms in both a TLR2-dependent and -independent manner.

[68] Pennini ME, Liu Y, Yang J, Croniger CM, Henry Boom W, Harding CV (2007). CCAAT/enhancer-binding protein *β* and *δ* binding to CIITA promoters is associated with the inhibition of CIITA expression in response to *Mycobacterium tuberculosis* 19-kDa lipoprotein. *Journal of Immunology*.

[69] Pulido I, Leal M, Genebat M, Pacheco YM, Sáez ME, Soriano-Sarabia N (2010). The TLR4 ASP299GLY polymorphism is a risk factor for active tuberculosis in caucasian HIV-infected patients. *Current HIV Research*.

[70] Das P, Lahiri A, Lahiri A, Chakravortty D (2010). Modulation of the arginase pathway in the context of microbial pathogenesis: a metabolic enzyme moonlighting as an immune modulator. *PLoS Pathogens*.

[71] Talaue MT, Venketaraman V, Hazbón MH (2006). Arginine homeostasis in J774.1 macrophages in the context of Mycobacterium bovis BCG infection. *Journal of Bacteriology*.

[72] El Kasmi KC, Qualls JE, Pesce JT (2008). Toll-like receptor-induced arginase 1 in macrophages thwarts effective immunity against intracellular pathogens. *Nature Immunology*.

[73] Qualls JE, Neale G, Smith AM (2010). Arginine usage in mycobacteria-infected macrophages depends on autocrine-paracrine cytokine signaling. *Science Signaling*.

[74] Spierings E, De Boer T, Wieles B, Adams LB, Marani E, Ottenhoff THM (2001). Mycobacterium leprae-specific, HLA class II-restricted killing of human Schwann cells by CD4^+^ Th1 cells: a novel immunopathogenic mechanism of nerve damage in leprosy. *Journal of Immunology*.

[75] Oliveira RB, Ochoa MT, Sieling PA (2003). Expression of toll-like receptor 2 on human schwann cells: a mechanism of nerve damage in leprosy. *Infection and Immunity*.

[76] Goethals S, Ydens E, Timmerman V, Janssens S (2010). Toll-like receptor expression in the peripheral nerve. *GLIA*.

[77] López M, Sly LM, Luu Y, Young D, Cooper H, Reiner NE (2003). The 19-kDa *Mycobacterium tuberculosis* protein induces macrophage apoptosis through toll-like receptor-2. *Journal of Immunology*.

[78] Robbins G, Mushrif Tripathy V, Misra VN (2009). Ancient skeletal evidence for leprosy in India (2000 B.C.). *PLoS ONE*.

[79] Hershkovitz I, Donoghue HD, Minnikin DE (2008). Detection and molecular characterization of 9000-year-old *Mycobacterium tuberculosis* from a neolithic settlement in the Eastern mediterranean. *PLoS ONE*.

[80] Barreiro LB, Ben-Ali M, Quach H (2009). Evolutionary dynamics of human toll-like receptors and their different contributions to host defense. *PLoS Genetics*.

[81] Enard D, Depaulis F, Crollius HR (2010). Human and non-human primate genomes share hotspots of positive selection. *PLoS Genetics*.

